# *PbrmiR397a* regulates lignification during stone cell development in pear fruit

**DOI:** 10.1111/pbi.12950

**Published:** 2018-06-21

**Authors:** Cheng Xue, Jia‐Long Yao, Meng‐Fan Qin, Ming‐Yue Zhang, Andrew C. Allan, De‐Fu Wang, Jun Wu

**Affiliations:** ^1^ Center of Pear Engineering Technology Research State Key Laboratory of Crop Genetics and Germplasm Enhancement Nanjing Agricultural University Nanjing Jiangsu China; ^2^ The New Zealand Institute for Plant & Food Research Limited Auckland New Zealand; ^3^ School of Biological Sciences University of Auckland Auckland New Zealand

**Keywords:** pear (*Pyrus*), stone cell, lignin, laccase genes (*LAC* genes), miR397a

## Abstract

Lignified stone cells substantially reduce fruit quality. Therefore, it is desirable to inhibit stone cell development using genetic technologies. However, the molecular mechanisms regulating lignification are poorly understood in fruit stone cells. In this study, we have shown that microRNA (miR) miR397a regulates fruit cell lignification by inhibiting laccase (*LAC*) genes that encode key lignin biosynthesis enzymes. Transient overexpression of *PbrmiR397a*, which is the miR397a of Chinese pear (*Pyrus bretschneideri*), and simultaneous silencing of three *LAC* genes reduced the lignin content and stone cell number in pear fruit. A single nucleotide polymorphism (SNP) identified in the promoter of the *PbrmiR397a* gene was found to associate with low levels of fruit lignin, after analysis of the genome sequences of sixty pear varieties. This SNP created a TCA element that responded to salicylic acid to induce gene expression as confirmed using a cell‐based assay system. Furthermore, stable overexpression of *PbrmiR397a* in transgenic tobacco plants reduced the expression of target *LAC* genes and decreased the content of lignin but did not change the ratio of syringyl‐ and guaiacyl‐lignin monomers. Consistent with reduction in lignin content, the transgenic plants showed fewer numbers of vessel elements and thinner secondary walls in the remaining elements compared to wild‐type control plants. This study has advanced our understanding of the regulation of lignin biosynthesis and provided useful molecular genetic information for improving pear fruit quality.

## Introduction

Pear is an important fruit crop that belongs to the *Pyrus* genus in the Rosaceae family and has been cultivated for more than two thousand years in China (Lombard and Westwood, 1987). At least 22 *Pyrus* species have been identified worldwide. Five of them, *P. bretschneideri*,* P. pyrifolia*,* P. sinkiangensis*,* P. ussuriensis* and *P. communis*, are major cultivated species (Vavilov, 1951). The first four are mainly cultivated in China and in several other Asian countries, while *P. communis* is mainly cultivated in western countries. According to the Food and Agriculture Organization of the United Nations, China produces more than 60% of pears worldwide and supplies approximately 15% of exported pear markets. However, Chinese pears have been exported at a price below the market average mainly because of their high content of stone cells compared with that of European and Japanese pears. Therefore, it is important to reduce the stone cell content of Chinese pears to meet the demands of international customers.

Stone cells constitute a type of brachysclereid and are formed from parenchyma cells by deposition of lignin and cellulose to form secondary‐thickened cell walls (Smith, 1935). Stone cells specifically accumulate in pear flesh and contribute to poor fruit quality. To date, the physiological and anatomical aspects of pear stone cells have been studied; the formation of stone cells is closely correlated with the biosynthesis, transfer and deposition of lignin in pear flesh (Martin‐Cabrejas *et al*., 2006). Pear stone cells contain mostly guaiacyl‐lignin (G‐lignin), a small amount of syringyl‐lignin (S‐lignin), but no p‐hydroxyphenyl lignin (H‐lignin) (Cai *et al*., 2010; Jin *et al*., 2013).

Lignin biosynthesis has been extensively studied in *Arabidopsis* and *Populus*, as lignin is a vital component for plant secondary cell walls, the structural integrity of stems, and resistance against diseases and pests (Wainhouse *et al*., 1990). Lignin is also a major hurdle in the paper, bioethanol and forage industries (Chen and Dixon, 2007; Gnansounou and Dauriat, 2005; Sarkanen, 1976). Different kinds of enzymes, including phenylalanine ammonia‐lyase, cinnamate‐4‐hydroxylase, cinnamyl‐alcohol dehydrogenase, cinnamoyl‐CoA reductase, hydroxycinnamoyl transferase and caffeoyl shikimate esterase (Vanholme *et al*., 2013; Zhao, 2016), are known to be involved in synthesizing three monolignols (G‐, S‐ and H‐type lignin) from phenylalanine. Lignin is a polymer of these three different monolignols (Higuchi, 2003). Recent structural characterization of cell walls from monocot species revealed that the flavone tricin was a part of the native lignin polymer in wheat (Río *et al*., 2012; Zeng *et al*., 2013), coconut coir (Rencoret *et al*., 2013), bamboo (*Phyllostachys pubescens*) (Wen *et al*., 2013), maize (*Zea mays*) (Lan *et al*., 2015) and sugarcane (*Saccharum officinarum*) (Río *et al*., 2015). The final dehydrogenative polymerization of monolignols into lignin is catalysed by either peroxidase (POD) or laccase (LAC) enzymes (Dean and Eriksson, 1994). The *ZePrx* encoding cationic POD enzyme catalyses the last step of lignification in *Zinnia elegans* (Gabaldón *et al*., 2005). In *Arabidopsis*, 73 *POD* genes have been identified as homologues of *ZePrx*, and the important role of *AtPrx72* gene in lignification has been confirmed (Herrero *et al*., 2013).

Plant LACs form a large family of oxidases that contain three conserved blue copper protein domains (Zhukhlistova *et al*., 2008). LACs may be involved in lignin polymerization in *Populus trichocarpa* and *Arabidopsis*. Five *LAC* genes (*LAC1*,* LAC2*,* LAC3*,* LAC90* and *LAC110*) have been cloned from the xylem of *P. trichocarpa* based on their expression during xylem lignification (Ranocha *et al*., 1999). A reduction in both secondary cell wall thickness and lignin content of xylem fibres was observed in transgenic *Populus* plants when *LAC3*,* LAC90* and *LAC110* were all suppressed by RNA interference. However, this type of reduction was not detected when the *LAC3* gene was suppressed alone (Ranocha *et al*., 1999). Eight of the seventeen *LAC* genes in *Arabidopsis* (Cai *et al*., 2006; McCaig *et al*., 2005) show an abundance of transcripts in lignified inflorescence stem tissue (Berthet *et al*., 2011; Turlapati *et al*., 2011). Disruption of *AtLAC4* and *AtLAC17* only leads to a slight change in lignin content in *Arabidopsis* (Berthet *et al*., 2011). Simultaneous disruption of *AtLAC4*,* AtLAC11* and *AtLAC17* causes severe arrest in both vascular and whole plant development, reduces root diameters and produces indehiscent anthers due to the reduction in lignification (Zhao *et al*., 2013). It is difficult to demonstrate separately the function of each *POD* or *LAC* gene because these genes are functionally redundant (Turlapati *et al*., 2011).

MicroRNAs (miRs) are small noncoding RNAs with a length of 19–24 nucleotides and play important roles in modulating gene expression (Brodersen and Voinnet, 2009). MiR397 is a conserved miR in flowering plants (Kozomara and Griffiths‐Jones, 2010) and was found to target *LAC* transcripts for cleavage in *Arabidopsis* (Wang *et al*., 2014) and *P. trichocarpa* (Lu *et al*., 2013). In rice, overexpression of miR397 increases rice grain size and panicle branching via modulating the expression of *OsLAC*, whose product is a LAC protein that is involved in plant sensitivity to brassinosteroids (Zhang *et al*., 2013).

A species‐specific regulation mechanism for lignin biosynthesis is likely present in some plants (Wang and Chiang, 2014). Different from model plants, fruit crop species may have specific mechanisms to minimize lignin biosynthesis and accumulation in fruit, as high fruit lignin levels affect fruit quality and economic value. However, until recently, little progress has been made in understanding these specific mechanisms. Fruit firmness and the lignin content of ‘Luoyangqing’ loquat (*Eriobotrya japonica*) increase during cold storage when a chilling injury occurs, and *EjMYB1*,* EjNAC1, EjAP2‐1* and *EjHSF3* were found to involve in lignification in the cold‐injured fruit by activating lignin biosynthesis genes (Xu *et al*., 2014, 2015; Zeng *et al*., 2015, 2016).

We previously showed that *PbrmiR397a* is abundantly expressed at an early stage of pear fruit development and potentially targets 27 pear *LAC* genes (Wu *et al*., 2014). The function of LACs in lignin biosynthesis in pear fruit is still unknown (Wu *et al*., 2013), although PODs are known to catalyse oxidative polymerization of monolignols into lignin polymers. In this study, we analysed the expression patterns and protein subcellular locations of pear *LAC* genes as well as the functions of miR397a and LACs during pear fruit stone cell development. The results suggest that *PbrLAC*s are functionally redundant and are negatively controlled by *PbrmiR397a* during stone cell development in pear fruit. Our research provides both new evidence to reveal the functions of miR397a and its target genes in regulating pear fruit stone cell formation and guidance for improving fruit quality by reducing stone cell content.

## Results

### Stone cell formation and lignin deposition mainly occur during early stages of pear fruit development

**Figure 1:**
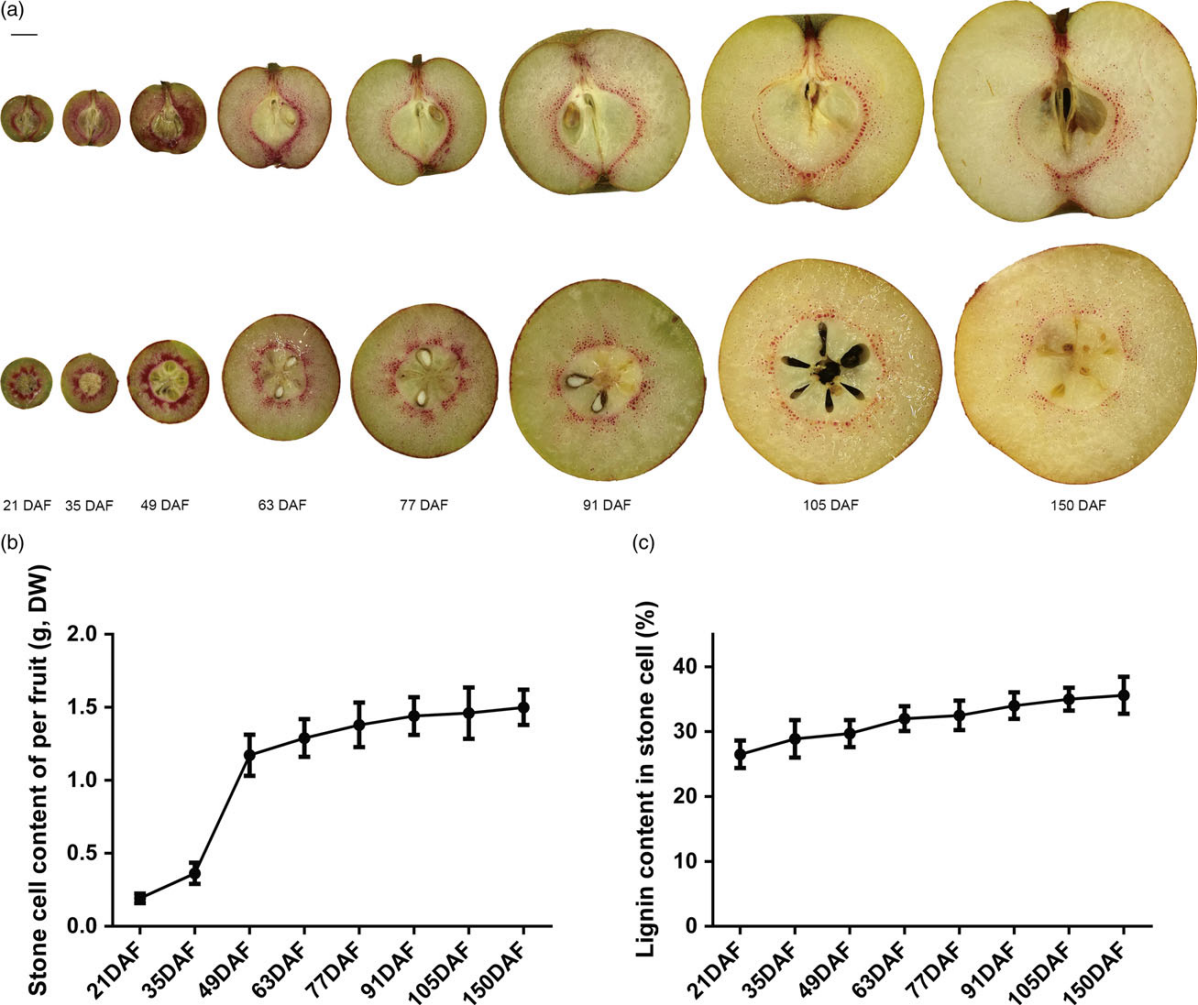
Hand‐cut sections, stone cells and lignin content of ‘Dangshansuli’ fruit at eight developmental stages. (a) The sections were stained by Wiesner's reagent. Longitudinal sections are in the top panel, and cross‐sections are in the bottom panel. Scale bar = 1 cm. DAF, days after full bloom. (b, c) Quantification of stone cell and lignin in ‘Dangshansuli’ fruit at eight developmental stages. DW, dry weight. The values are the mean ± SE of three independent experiment repeats.

To examine lignin deposition in the flesh of pear fruit, hand‐cut sections were stained with phloroglucinol‐HCl (Wiesner reagent). As shown in Figure 1a, lignified stone cells were stained red violet. The staining pattern indicates that stone cells formed rapidly from 21 to 49 days after full bloom (DAF), after which their distribution became gradually diluted from 63 to 150 DAF (Figure 1a). Stone cell content accumulated rapidly in fruit from 21 to 49 days, remaining stable after 49 DAF (Figure 1b). In addition, lignin content in stone cells remained a stable level at each development stages (Figure 1c). In general, stone cell formation and lignin deposition in pear fruit occur during the early stages of fruit development. Stone cells and lignin will not be degraded after their formation but be maintained in fruit flesh.

### Genomewide identification of *LAC* genes and correlation of *LAC*s with lignin biosynthesis during pear fruit development

**Figure 2:**
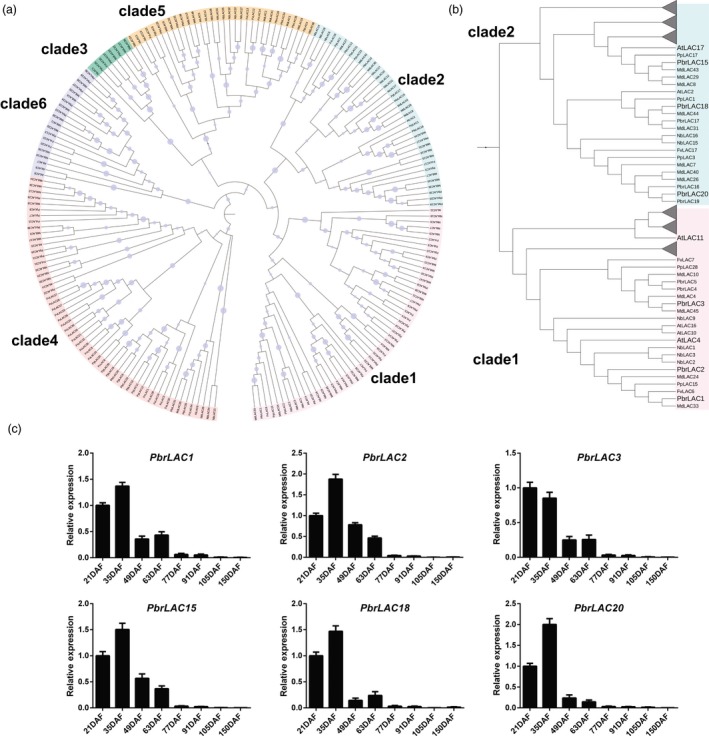
Phylogenetic relationships of LACs and relative expression levels of *PbrLAC*s. (a) Phylogenetic relationships of 38 PbrLACs, 48 MdLACs, 31 PpLACs, 37 FvLACs, 34 NbLACs and 17 AtLACs. Six clades are indicated by clades 1–6. (b) Enlarged clades of LAC proteins from clades 1 and clade 2, and selected branches collapsed as triangles for clarity. Six candidate PbrLACs and three lignin biosynthesis AtLACs are shown in bold type on the tree for emphasis. (c) Relative expression of *PbrLAC*s at the same fruit developmental stages as in Figure 1.

Based on the results of Basic Local Alignment Search Tool (BLAST) queries, 38, 48, 31, 37 and 34 *LAC* genes were identified from pear, apple, peach, strawberry and tobacco genomes, respectively. These homologues were named as *PbrLAC*s, *MdLAC*s, *PpLAC*s, *FvLAC*s and *NbLAC*s (Table S1). Based on the results of phylogenetic analyses, *LAC*s were grouped into six clades, clades 1–6 (Figure 2a). Eleven *PbrLAC*s clustered with *AtLAC4*,* AtLAC10*,* AtLAC11* and *AtLAC16* in clade 1 and nine clustered with *AtLAC17* in clade 2 (Figure 2b). It was previously reported that mutations of *AtLAC4*,* AtLAC11* and *AtLAC17* led to a lignin content reduction, while the mutations of *AtLAC10* and *AtLAC16* caused no altered phenotypes in *Arabidopsis* (Berthet *et al*., 2011; Cai *et al*., 2006; Zhao *et al*., 2013). We predicted that some of *PbrLAC* genes might be involved in lignin biosynthesis in pear fruit as they were clustered together with *AtLAC4*,* AtLAC11* and *AtLAC17*.

RNA‐Seq data of six pear cultivars (‘Dangshansuli’, ‘Hosui’, ‘Yali’, ‘Kuerlexiangli’, ‘Nanguoli’ and ‘Starkrimson’) were generated at six fruit development stages (beginning of fruit set, physiological fruit drop, fruit rapid enlargement, a month after fruit enlargement, pre‐mature and mature stage) (Wu *et al*., 2013; Zhang *et al*., 2016). From these data, the transcripts were detected for 29 of the 38 *PbrLAC* genes. Six of these genes (*PbrLAC1*,* 2*,* 3*,* 15*,* 18* and *20*) were abundantly expressed during the early stage of fruit development while other genes were very weakly expressed and excluded from further studies (Table S2). In apple, none of the *MdLAC*s were abundantly expressed during fruit flesh development (Table S3). In peach and strawberry, only *PpLAC11* and *FvLAC1*, which belong to clade 6 in the phylogenetic tree, were expressed abundantly during fruit development (Tables S4 and S5) (Gu *et al*., 2016; Kang *et al*., 2013). However, in that clade, no genes were reported to be directly involved in lignin biosynthesis. The six *PbrLAC* genes were selected to verify their expression at eight different stages of fruit development for ‘Dangshansuli’ by q‐PCR. All six genes were expressed abundantly at 21 and 35 DAF, prior to lignin content peaking at 49 DAF, after which expression decreased rapidly (Figure 2c). The expression levels of *PbrLAC* genes were consistent with lignin contents during fruit development. Based on the phylogenetic tree and expression analysis, these *PbrLAC* genes are candidates for catalysing lignin biosynthesis in the formation of stone cells (the specific trait that differs from the other three Rosaceae species) in pear fruit.

To examine the subcellular localization of these pear LAC proteins, *PbrLAC*‐*GFP* expression vectors of *PbrLAC1*,* 2* and *18*, which had higher expression during fruit development according to the RNA‐Seq data (Table S2), were transferred into onion epidermal cells. In plasmolysed cells, green fluorescence signals from the fusion constructs *PbrLAC1*‐*GFP*,* PbrLAC2*‐*GFP* and *PbrLAC18*‐*GFP* were detected specifically in cell walls, and signals from the 35S‐GFP control construct were detected throughout the cell (Figure S1). This result suggests that these LAC proteins are specifically located in cell wall and are colocated at the site of lignin biosynthesis.

### Verification of *PbrLAC*s and *NbLAC*s as targets of *PbrmiR397a*

**Figure 3:**
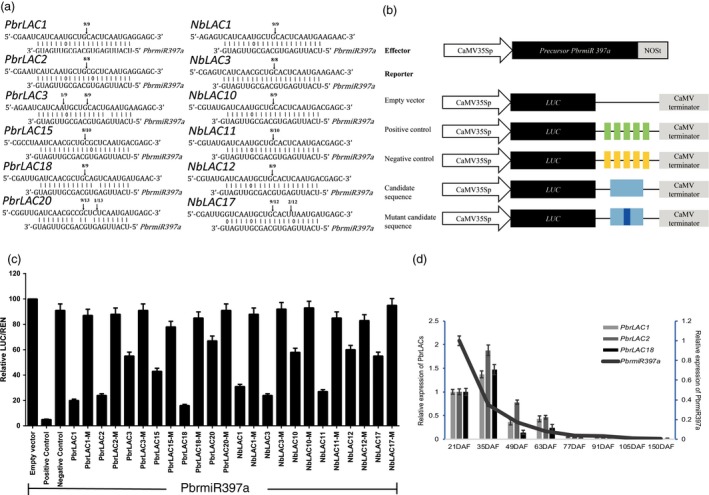
*PbrmiR397a* targets *PbrLAC*s and *NbLAC*s for cleavage and suppression of expression during fruit development. (a) Experimentally validated targets of *PbrmiR397a*. The cleavage sites were determined by the modified 5′‐RNA ligase‐mediated RACE. The *PbrLAC* sequence of each complementary site from 5′ to 3′ and the *PbrmiR397a* sequence from 3′ to 5′ are shown. Watson–Crick pairing (vertical dashes) and G:T wobble pairing (circles) are indicated. Vertical arrows indicate the 5′‐termini of miRNA‐guided cleavage products, as identified by 5′‐RACE, and the frequency of clones is shown. (b) Schematic diagram of gene constructs. Constructs of 35s::precursor *PbrmiR397a* used as exogenous source of *PbrmiR397a*. In reporter vector, five copies of a perfect *PbrmiR397a* target sequence (5 ×  miR397‐ts) which was fused immediately after the LUC stop codon as positive control. And five copies of inverted *PbrmiR397a* target sequence (5 ×  miR397‐its) was used as negative control. The candidate target sequence of *PbrmiR397a* was PCR amplified from cDNA. And mutant candidate target was altered candidate target sequence at cleavage sites. (c) Relative activity levels of luciferase. Mis, mismatch mutant of candidate target sequence. The data are mean of relative ratio of LUC/REN (*n* = 6), and the error bars represent standard deviation (SD). (d) Relative expression of *PbrmiR397a* at eight fruit development stages. Bar charts indicate relative gene expression levels of *PbrLAC*s, and line charts indicate those of *PbrmiR397a*.

We have previously shown that 27 *LAC* genes identified from genomic sequences are potential target genes of *PbrmiR397a* (Wu *et al*., 2014). In this study, 20 *NbLAC*s were identified as potential targets of miR397a in tobacco. The target sites of *PbrmiR397a* are located in sequences encoding a conserved Cu‐oxidase domain in both pear and tobacco genes (Figure S2a). We performed a 5′‐rapid amplification of cDNA ends (5′‐RACE) analysis (Leng *et al*., 2016) using six *PbrLAC*s (*1*,* 2*,* 3*,* 15*,* 18* and *20*) selected based on their strong expression in pear fruit and six tobacco *NbLAC*s (*1*,* 3*,* 10*,* 11*,* 12* and *17*). The analysis verified that all 12 *LAC*s were targets of *PbrmiR397a* (Figure 3a). To assay targeting, we constructed a vector containing the miR397a gene and a series of reporter vectors containing firefly luciferase (*LUC*) gene fused to a miR397a target sequences or a mutated target sequences amplified from *LAC* genes (Figure 3b). When young *Nicotiana benthamiana* leaves were infiltrated with a reporter vector only, LUC activities were similar among leaves infiltrated with the control reporter or with a test reporter (Figure S3). This result suggests that there was little endogenous miR397a in *N. benthamiana* leaves. When leaves were infiltrated with a reporter vector together with the miR397a effector vector, LUC activities were significantly lower in leaves infiltrated with LUC reporter fused to a miR397a target sequence than those in leaves infiltrated with LUC reporter fused to a mutated miR397a target sequence (Figure 3c). This result indicated that miR397a might act on the target sequence but not the mutated target sequence to regulate the expression of LUC. The results of both 5′‐RACE and reporter gene analysis verified the computational prediction and supported a regulatory role of *PbrmiR397a* in suppressing these *LAC*s.

The 24 *PbrLAC*s targeted by *PbrmiR397a* belonged to clades 1, 2, 3, 4 and 6 (Figure 2a), but none of them were classified into clade 5. The relative expression of *PbrmiR397a* was abundant at 21 DAF but decreased rapidly thereafter. *PbrmiR397a* expression contrasted with that of *PbrLAC*s at 21 and 35 DAF, but the expression of both decreased rapidly thereafter (Figure 3d). This suggests that *PbrmiR397a* plays an important role by regulating the expression of *PbrLAC*s in the early stages of fruit development.

### Overexpression of *PbrmiR397a* or the simultaneous silencing of *PbrLAC1*,* 2*, and *18* reduces lignin contents in pear fruits

**Figure 4:**
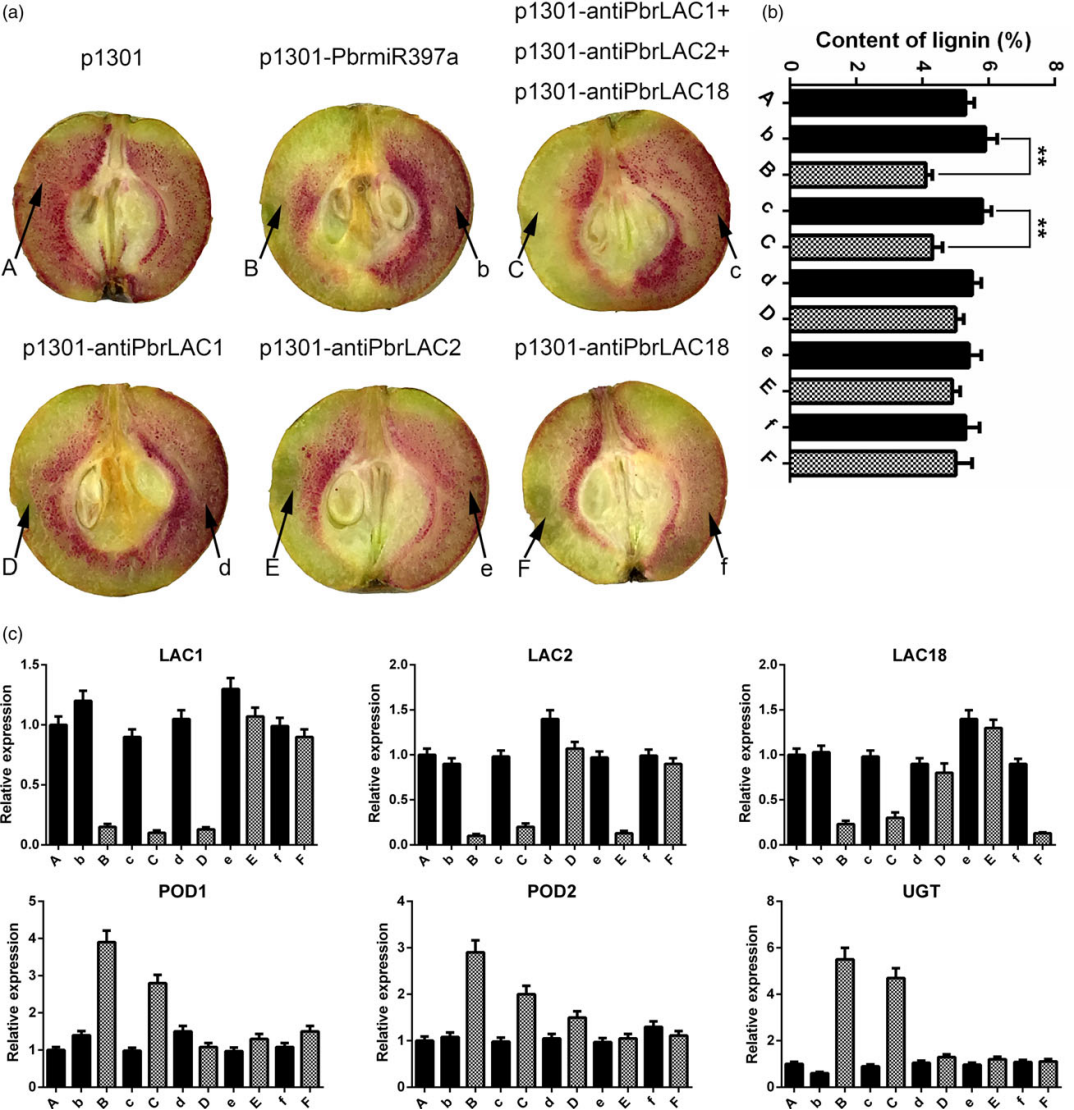
Lignin biosynthesis and gene expression patterns in fruit tissue infiltrated with *PbrmiR397a* overexpression and *PbrLAC* antisense constructs. (a) Transient assays using *PbrmiR397a* overexpression and *PbrLAC1*,* PbrLAC2* and *PbrLAC18* antisense constructs in ‘Dangshansuli’ fruit at 35 DAF. The infiltration sites are labelled A, B, C, D, E and F for the different gene constructs, and their respective noninfiltrated sites are labelled b, c, d, e and f, respectively. The image was taken 10 days after agroinfiltration. (b) Lignin content in the fleshy tissue around the infiltration sites (A, B, C, D, E and F) and their respective noninfiltrated sites (b, c, d, e and f). ‘**’ represents highly significant difference according to *t*‐test (*P* < 0.01). Error bars indicate SE from three independent experiments. (c) Expression levels of the *PbrLAC*s, *PbrPOD*s and *PbrUGT72E* in the fleshy tissue around the infiltration sites and their respective noninfiltrated sites using q‐PCR analysis.

To further elucidate the roles of *PbrmiR397a* and its targets *PbrLAC1*,* 2* and *18* in lignin biosynthesis, miR397a overexpression or *LAC* antisense constructs were agroinfiltrated into ‘Dangshansuli’ pear fruit at 35 DAF. Ten days after infiltration, a strong reduction in lignin staining was observed at infiltration sites both for miR397a overexpression and for LAC1, 2, and 18 silencing together, but a reduction in lignin staining was not observed at the injection sites for the control vector or for single *LAC* antisense constructs (Figures 4a and S4).

The lignin content in the fleshy tissue around the infiltration sites showed 30.5% and 25.9% reduction for 35S‐*PbrmiR397a* and 35S‐anti*PbrLAC1*,* 2*,* 18* together, respectively, when compared with that of the corresponding noninfiltrated sites (Figure 4b). The lignin content at other sites infiltrated with 35S‐anti*PbrLAC1*, 35S‐anti*PbrLAC2* or 35S‐anti*PbrLAC18* decreased moderately. However, no significant differences were observed compared with that of their corresponding noninfiltrated sites. In addition, the lignin content in the fleshy tissue around the infiltration sites was unchanged when the control empty vector (EV) was used.

The expression of six genes (*PbrLAC1*,* PbrLAC2*,* PbrLAC18*,* PbrPOD1*,* PbrPOD2* and *PbrUGT72E*) at the injection sites was analysed by q‐PCR. *PbrPOD1*,* PbrPOD2* and *PbrUGT72E* are putative orthologous genes involved in the transfer of monolignols in *Arabidopsis* (Lin *et al*., 2016). Injection of the *PbrmiR397a* overexpression construct decreased the expression of *PbrLAC1*,* PbrLAC2* and *PbrLAC18* but increased the expression of *PbrPOD1*,* PbrPOD2* and *PbrUGT72E*. This gene expression pattern was repeated after simultaneously injecting the *PbrLAC1*,* 2* and *18* silencing constructs (Figure 4c). However, injection of each individual *PbrLAC* gene‐silencing construct suppressed only its own expression and not the expression of the other five genes in the lignin pathway.

### Sequence and expression analyses of *PbrmiR397a* and *PbrLAC*s in pear varieties reveal the basis of stone cell content

**Figure 5:**
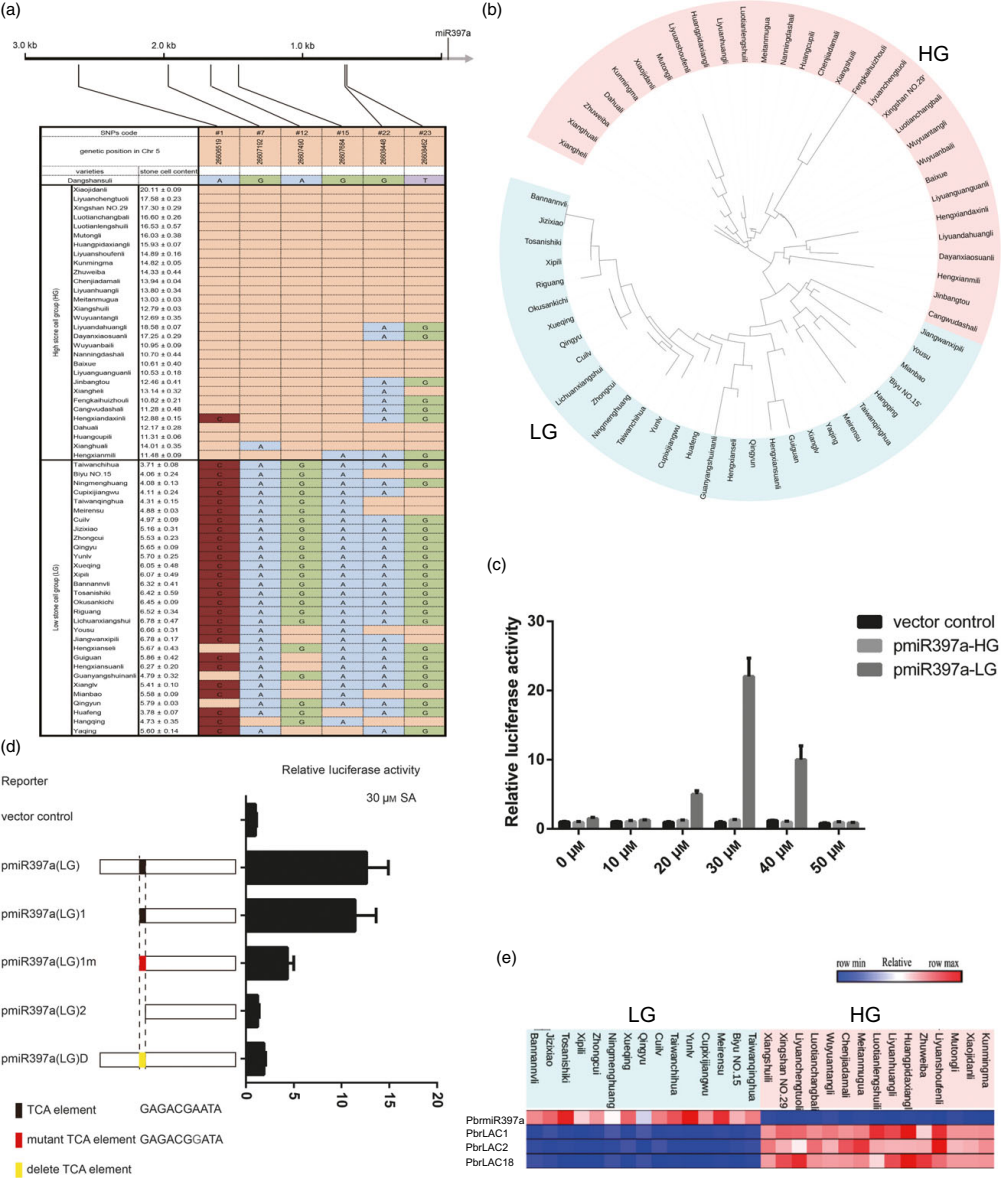
Gene sequence and expression analysis of pear varieties that differ in stone cell content. (a) DNA polymorphisms in the *PbrmiR397a* promoter region among 60 pear varieties compared with the ‘Dangshansuli’ reference sequence. Blanks indicate the same nucleotide as in ‘Dangshansuli’. (b) Phylogenetic analysis was performed on the nucleotide sequence of the 3.0‐kb promoter region of *PbrmiR397a* in the 60 varieties. (c) Results of the protoplast transient assay. Relative activity levels of luciferase driven by the 3.0‐kb *PbrmiR397a* promoter fragments from the HG and LG. Each sample was treated with a series of concentrations of SA. (d) Relative activity levels of luciferase driven by different *PbrmiR397a* promoter fragments from the LG. The scheme for the deletion variants of the *PbrmiR397a* promoter is shown on the left. (e) Expression of *PbrmiR397a* and three *PbrLAC*s in the fruit of selected 30 pear varieties at 35 DAF. Heatmap of expression levels of *PbrmiR397a*,* PbrLAC1*,* PbrLAC2* and *PbrLAC18*.

The stone cell content of 304 pear varieties has been assessed (Zhang *et al*., 2016). Of these varieties, 30 high‐ and 30 low‐stone cell‐content varieties were selected to evaluate the relationship between stone cell content and genetic variation in *PbrmiR397a* and *PbrLAC*s. The average stone cell content ranged from 10.53% to 20.11% for the high group (HG) and from 3.71% to 6.78% for the low group (LG) (Figure 5a). After sequencing the genome of these 60 pear varieties, we identified SNPs in the genomic region of *PbrmiR397a* (3107 bp), *PbrLAC1* (4985 bp), *PbrLAC2* (4943 bp), and *PbrLAC18* (4822 bp). There was no correlation between the SNPs of *PbrLAC*s and stone cell content (Table S6). Twenty‐five SNPs (named #1 to #25) were identified in the 3‐kb promoter region of the *PbrmiR397a* gene among the sixty pear varieties, but none were identified in the *PbrmiR397a* precursor. Of these 25 SNPs, four (#1, #7, #12 and #15) were highly correlated with stone cell content in the HG and LG (Figure 5a). A phylogenetic tree based on the upstream sequences of *PbrmiR397a* classified all 30 HG varieties into one clade and all 30 LG varieties into another clade (Figure 5b), which indicated that these four SNPs are useful for marker‐assisted breeding. To verify the identified SNPs, the 3‐kb promoter region of *PbrmiR397a* was cloned from three randomly selected varieties in both the HG and LG. The complete sequencing results of these 3‐kb fragments confirmed all previously identified SNPs and revealed six new SNPs, but the results did not reveal any indels. These six new SNPs did not correlate with stone cell content (Figure S5).

Using the PlantCARE database for analyses of conserved promoter elements (Rombauts *et al*., 1999), we identified several known cis‐elements in the promoter region of *PbrmiR397a* in the HG and LG. SNP #7 (G>A) creates a TCA element, and SNP #15 (G>A) disrupts a TGACG motif in the LG varieties. All other identified cis‐elements were the same between the HG and LG groups. The TCA element and TGACG motif are hormone‐responsive elements. To determine whether the SNPs affect promoter activity, dual‐luciferase reporter assays were performed using *Arabidopsis* leaf protoplasts. The assay results showed that the levels of luciferase driven by pmiR397a‐HG and pmiR397a‐LG were low and did not respond to auxin treatments (Figure S6). However, application of 20, 30 or 40 μm salicylic acid (SA) greatly enhanced the levels of luciferase driven by pmiR397a‐LG but not by pmiR397a‐HG (Figure 5c). To determine whether the alteration in cis‐elements caused by SNP #7 is essential for the SA‐inducible luciferase activity, deletion variants of the pmiR397a‐LG promoter were tested using the assay. The promoter fragments pmiR397a‐LG and pmiR397a‐LG1, both containing a TCA element, were activated by treatment with 30 μm SA. Other promoters that lack the TCA element or that contain the mutant TCA element displayed low levels of luciferase activity (Figure 5d). In pear fruit, the expression level of *PbrmiR397a* was significantly lower in the 15 HG varieties than in 15 LG varieties. In contrast, the expression level of *PbrLAC*s was higher in the 15 HG varieties than in the 15 LG varieties (Figure 5e). Based on these results, we propose that the alterations to cis‐elements caused by SNP #7 may be responsible for the different expression levels of *PbrmiR397a* between the HG and LG varieties.

### Overexpression of *PbrmiR397a* reduces *NbLAC* transcript levels and lignin content in transgenic tobacco plants

**Figure 6:**
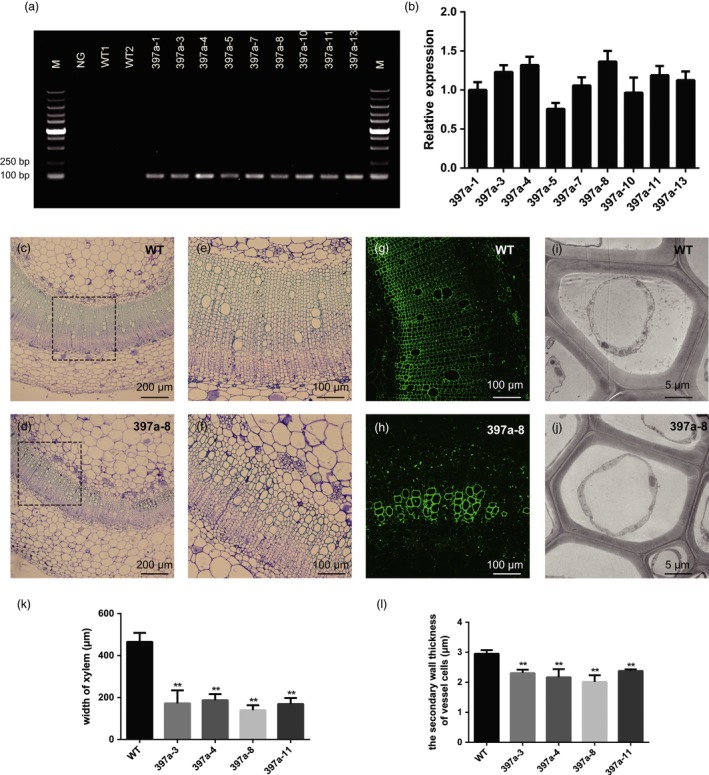
Overexpression of *PbrmiR397a* in transgenic tobacco plants. (a) DNA amplification of *PbrmiR397a* precursor sequence in WT and nine hygromycin‐resistant primary transformants. M, DNA marker. NG, negative control. (b) Expression of *PbrmiR397a* in T2‐generation transgenic plants. (c–f) Stem cross‐sections of 397a‐8 (d, f) showed fewer vessel elements than did the sections of WT plants (c, e). (e) and (f) are higher magnifications of the areas marked by dotted lines in (c) and (d), respectively. (g and h) Lignin autofluorescence (green colour) decreased in the vascular bundles and interfascicular fibres of the 397a‐8 transgenic line (h) compared with WT (g). (i and j) Transmission electron micrographs of vessels. The secondary cell walls of vessel elements were thinner in the 397a‐8 transgenic line (j) than in WT (i). (k and l), Statistical analysis of width of xylem and the secondary cell thickness of vessel cells in WT and T2 transgenic plants. Data are mean of five plants (*n* = 5), the error bars representing standard error. Asterisks indicate values that were determined by the *t*‐test to be significantly different from their equivalent control *P* < 0.01 (**). The second highest internode of 8‐week‐old tobacco plants were used for the assay, and five plants for the WT and T2‐generation *PbrmiR397a* transgenic lines were used.

Nine hygromycin‐resistant primary transgenic plants were confirmed to contain the transgene *PbrmiR397a* (Figure 6a). These plants were grown to T2 generation and used for q‐PCR analysis to determine the expression of *PbrmiR397a*. Four transgenic lines (397a‐3, 397a‐4, 397a‐8 and 397a‐11) that exhibited relatively high levels of *PbrmiR397a* expression (Figure 6b) were selected for further analyses in T2 generation. The biomass measurements, including plant height, leaf area, and fresh weight of leaves and whole plant of T2 homozygous transgenic tobacco showed no significant differences from that of wild‐type (WT) control plants (Table 1). However, the T2 plants showed 18%–29% reduction in lignin content compared to WT control plants (Table 2). Even with this reduction, there was no change in the ratio of S‐/G‐lignin monomers compared to WT plants (Table 2).

**Table 1:** Biomass measurements of WT and *PbrmiR397a* transgenic tobacco plants

Line	Height of stem (m)	Total leaf area per plant (cm^2^)	Biomass	
			Leaf weight per plant (kg, FW)	Whole plant weight (kg, FW)
WT	1.32 ± 0.07	22 969 ± 1173	1.71 ± 0.27	2.96 ± 0.25
397a‐3	1.37 ± 0.04	21 146 ± 2142	1.72 ± 0.13	2.61 ± 0.19
397a‐4	1.27 ± 0.06	17 950 ± 2851	1.57 ± 0.13	2.63 ± 0.21
397a‐8	1.32 ± 0.08	19 264 ± 1199	1.75 ± 0.12	2.50 ± 0.18
397a‐11	1.26 ± 0.05	20 602 ± 666	1.51 ± 0.15	2.72 ± 0.12

Data are shown as mean ± SD of eight WT or T2 transgenic plants (*n* = 8) at 16 weeks old. The means were not significant difference between transgenic and WT plants as determined by *t*‐test. FW, fresh weight.

**Table 2:** Lignin content and composition for WT and *PbrmiR397a* transgenic tobacco plants

Line	Klason lignin content (%)	Thioacidolysis yield in μmol/g of extract‐free sample			S/G ratio
		G	S	Total G and S unit	
WT	22.8 ± 0.6 (100%)	125.2 ± 2.0	139.2 ± 1.9	264.4 ± 3.6	1.11 ± 0.01
397a‐3	18.6 ± 0.5** (82%)	111.6 ± 2.9**	127.3 ± 1.3**	238.8 ± 4.0**	1.14 ± 0.02
397a‐4	18.5 ± 0.6** (81%)	113.0 ± 3.3*	128.3 ± 1.2**	241.3 ± 4.2**	1.14 ± 0.03
397a‐8	16.3 ± 0.4** (71%)	102.5 ± 2.5**	118.2 ± 2.2**	220.7 ± 4.5**	1.15 ± 0.01
397a‐11	18.0 ± 0.4** (79%)	106.3 ± 2.7**	120.6 ± 3.0**	226.9 ± 3.6**	1.14 ± 0.05

Data are shown as mean ± SD (*n* = 4) of four independent experiments that used pooled xylem tissues of five WT or T2 transgenic plants at 16 weeks old. Asterisks indicate values that were determined by the *t*‐test to be significantly different from their equivalent control [*P* < 0.05 (*) and *P* < 0.01 (**)].

These tobacco lines also showed a different arrangement of vascular tissue; the vascular tissue had highly reduced numbers of vessel elements (Figure 6c–f) and thinner xylem regions (Figure 6k). Ultraviolet (UV) autofluorescence further confirmed that lignin autofluorescence was stronger in the xylem tissues of WT than in those of the 397a‐8 line (Figure 6g,h). The secondary wall thickness of transgenic *PbrmiR397a* lines was also significantly lower than that of WT plants (Figure 6i,j and l).

The expression of fifteen of the 20 *LAC* genes that are targets of *PbrmiR397a* in *Nicotiana tabacum* in stem‐differentiating xylem (SDX) of the second internode of 2‐month‐old tobacco plants was measured by q‐RT‐PCR analysis (Figure S7). Nine of the 15 SDX‐expressed *LAC*s were down‐regulated by 28%–90% in the *PbrmiR397a* transgenic line compared with the WT. Nontarget genes of miR397a, which included *POD*,* PRX* and *UGT72E*, were up‐regulated more than twofold. There was no significant change in expression of other genes tested (Table S7), which indicated that the reduced lignin content is specifically caused by the down‐regulation of the nine *LAC*s.

## Discussion

### Manipulation of *miR397* alters lignin content

In rice, overexpression of *miR397* promotes increased branch numbers and increased grain size via the suppression of *OsLAC* (Zhang *et al*., 2013). In poplar, stem lignin content is reduced in transgenic poplar plants overexpressing *PtrmiR397a* (Lu *et al*., 2013). In *Arabidopsis*, overexpression of *miR397b* resulted in normal plant development but reduced lignin content, increased number of seeds and enlarged seed size (Wang *et al*., 2014). In the present study, overexpression of *PbrmiR397a* in tobacco plant led to reduced lignin content; however, no developmental effects were observed. In contrast, disruption of *AtLAC4* and *AtLAC17*, two target genes of miR397, leads to a semidwarf phenotype that has reduced lignin content under conditions of continuous light (Berthet *et al*., 2011); triple mutants (*AtLAC4*,* AtLAC11* and *AtLAC17*) are severely dwarfed and exhibit both very low lignin levels and severely collapsed xylem cells (Zhao *et al*., 2013). Overexpression of *OsLAC* in WT plants results in arrested plant growth, reduced survival rates, and reduced grain yield and biomass (Zhang *et al*., 2013). All these results suggest that manipulation of *miR397* might be a more effective way for reducing lignin biosynthesis than manipulation of individual *LAC* genes, as *miR397* can regulate the transcript levels of several *LAC* genes simultaneously without causing apparent adverse effects on plant development.

### Plant hormones may regulate the expression level of *miR397*

In pear, *PbrmiR397a* expression levels were significantly higher in the varieties with low levels of stone cells (LG), compared with high stone cell (HG) varieties, whereas the expression levels of *PbrLAC*s were opposite to those of *PbrmiR397a*. A strong association between high levels of *PbrmiR397a* and low levels of lignin and stone cell content in different pear varieties was revealed. A SNP in the promoter of the *PbrmiR397a* gene was identified by genomewide sequencing to be associated with stone cell content in sixty pear varieties. In addition, we found that the SNP created a TCA element that may respond to SA signals and induce the expression of *PbrmiR397a*. In fact, most plant hormones affect the lignin biosynthesis pathway. Auxin negatively regulates vessel lignification during peach endocarp lignification (Zhang *et al*., 2015). Also, decreased levels of cytokinin in response to the reduced expression of *ABCG14*, a newly identified cytokinin transporter, lead to delayed lignin biosynthesis and reduced numbers of lignified cells (Zhang *et al*., 2014). Our results showed that alterations to cis‐elements caused by SNP #7 might result in the markedly different expression of *PbrmiR397a* observed between the HG and LG. This finding will help to develop DNA markers to detect pear varieties or progenies that have low lignin and stone cell contents.

### Relationships of *PbrLAC*s, *PbrPOD* and *PbrUGT72E* with lignin synthesis in pear fruit

LACs have similar structural conservation between bacteria, fungi and plants (Dwivedi *et al*., 2011). However, the number of *LAC* genes has increased; throughout plant evolution, the number of *LAC*s ranges from three in *Chlamydomonas reinhardtii* to 12 in *Physcomitrella patens*, 10 in *Selaginella moellendorffii* and 17–39 in angiosperms (Weng and Chapple, 2010). These numbers indicate that *LAC* members have expanded during the evolution from lower plant species to higher plant species. In *Arabidopsis*, the *lac4* and *lac17* double‐knockout mutant shows semidwarf phenotype and lower levels of lignin content under continuous light conditions (Berthet *et al*., 2011). Triple mutants of *AtLAC4*,* AtLAC11* and *AtLAC17* have significant lignin reduction and blocked development (Zhao *et al*., 2013), which indicates that all three *LAC* genes contribute to lignin synthesis and are functionally redundant. Our results reveal that the involvement of LACs during stone cell lignification is supported by specific LAC transcription profiles: expression levels of *PbrLAC* genes were positively correlated with lignin contents during fruit development and were higher in the HG varieties than in the LG varieties. Furthermore, the simultaneous silencing of *PbrLAC1*,* PbrLAC2* and *PbrLAC18* significantly reduced lignification, but silencing one *LAC* gene led to only a slight reduction in lignin content, which indicates functional redundancy among *PbrLAC* genes.

Up‐regulation of *POD* and *UGT72E* expression was observed in pear fruit cells when *PbrmiR397a* was overexpressed or when *PbrLAC*s were simultaneously silenced. To prevent the accumulation of monolignols in plasma membranes or inner cell walls when oxidative capacity is diminished, some form of feedback regulatory mechanism may exist (Vanholme *et al*., 2013). In *Arabidopsis*, the glycosyltransferases *UGT72E2* and *UGT72E3* synthesize glucosylate monolignols into coniferin or syringin (Lanot *et al*., 2006). Monolignol glycosylation could increase the solubility and decrease the reactivity of monolignols, and the up‐regulation of *PbrUGT72E* could be a detoxification mechanism.

Reverse genetic approaches have shown an active role of *POD*s during lignin formation in multiple species. The down‐regulation of *PRX60* in tobacco plants (Blee *et al*., 2003) or of *PRX3* in aspen (Li *et al*., 2003) results in a reduction of lignin accumulation and altered lignin composition. Moreover, *Arabidopsis AtPRX2*,* AtPRX71* and *AtPRX25* single‐ or double‐knockout mutants exhibit reduced lignin accumulation but normal stem height (Shigeto *et al*., 2013). However, we observed that lignin content was not recovered by the twofold up‐regulation of *POD* expression after *LAC* genes were repressed in pear fruit. This observation indicated that LAC and POD activities are nonredundant and that both are necessary for monolignol polymerization during lignin accumulation. This finding subsequently led to a hypothetical model in which LACs and PODs act in sequential order during cell lignification, starting with LACs and followed by PODs, to achieve full cell wall lignification. Once the initial lignin polymers are catalysed by *LAC*s, the late intervention of *POD*s is further supported, as *POD*s can form rigid crosslinks between lignin, extensins and hemicelluloses in the secondary cell wall (Lagrimini *et al*., 1987; Passardi *et al*., 2004).

The results of this research will aid future studies to reveal the regulatory network of lignin biosynthesis. Revealing of the regulatory network not only will help to improve pear fruit quality but also will promote the production of wood and biofuels.

## Experimental procedures

### Plant materials

‘Dangshansuli’ (*P. bretschneideri*, white pear group) fruit samples were collected from the same trees at 21, 35, 49, 63, 77, 91, 105 and 150 DAF in an orchard (Gaoyou County, Jiangsu Province, China). The fruit of 60 varieties was collected at 35 DAF from the Chinese National Pear Germplasm Repository in Wuhan.

### Histology

Hand‐cut sections of pear fruit were treated with 30% HCl (v/v) for 1 min, stained in 12% phloroglucinol and 95% ethyl alcohol (w/v) for 7 min, and then washed with water. Cell walls containing lignin can be stained by phloroglucinol‐HCl (Wiesner reagent); however, nonlignified cells are not clearly stained (Wan *et al*., 2014; Zhong *et al*., 2000). Pear fruit sections were imaged using a hand‐held camera.

### Analysing stone cell and lignin contents in fruit flesh

Stone cell content was carried out using frozen‐HCl treatment as previously described (Tao *et al*., 2009), and data were shown as dry weight (g) per pear fruit. The acetyl bromide‐based method was used to detect lignin in fruit. The lignin content was shown as the percentage (calculated lignin content/stone cells dry weight × 100). The methods were the same as those previously described (Tao *et al*., 2009). Three independent experiments were performed (at least ten fruits were used in each experiment).

### *LAC* gene identification and phylogenetic analysis

PbrLACs, MdLACs, PpLACs, FvLACs and NbLACs were identified by the comparison of 17 *Arabidopsis* LAC protein sequences against the predicted protein sequences of pear, apple, peach, strawberry and tobacco using the tBLASTn and BLASTp algorithms (Gertz *et al*., 2006) and an e‐value cut‐off of 1E^−10^. The BLAST results detected copper‐oxidase domains in National Centre for Biotechnology Information (NCBI) conserved domain database. A phylogenetic tree was constructed using MEGA 5.1 (Tamura *et al*., 2011). The reliability of branching was assessed by bootstrap resampling using 1000 replicates.

### Subcellular localization of PbrLACs

The full‐length cDNAs of *PbrLAC1*,* 2* or *18* were fused in frame to the N‐terminus of GFP cDNA to form fusion vectors 35S‐PbrLAC1‐GFP, 35S‐PbrLAC2‐GFP or 35S‐PbrLAC18‐GFP (Figure S8a). The fusion constructs and the control vector (35S‐GFP) were transferred into *Agrobacterium tumefaciens* strain GV3101 by the freeze–thaw method. The constructs were introduced into onion epidermal cells by *Agrobacterium*‐mediated transformation in accordance with previously described methods (Sun *et al*., 2007). One day after transformation, the onion epidermal cells were plasmolysed with a 0.3 g/mL sucrose solution and examined for green fluorescence signals using a Leica TCs SP2 spectral confocal microscope (Leica Microsystems, Wetzlar, Germany). To obtain comparable images, laser intensity, pinhole and photomultiplier gain settings were kept constant between samples.

### RNA and DNA isolation

Total RNA and DNA were extracted using the cetyl‐trimethylammonium bromide (CTAB)‐based method (Porebski *et al*., 1997). The RNA and DNA samples were quantified with a Nanodrop spectrophotometer (Thermo Fisher Scientific, Massachusetts, USA).

### Expression profiles of genes

First‐strand cDNA synthesis was performed using the SYBR PrimeScript miRNA RT‐PCR Kit (Takara, Kusatsu, Japan). Q‐PCR was performed using the LightCycler 480 (Roche, Basel, Switzerland). Each gene was repeated for three biological samples and three technical repeats. Relative expression levels of each gene were calculated using the 2^−ΔΔCp^ algorithm. *PbrGAPDH* and *U6* were used as reference genes for *PbrLACs* and *PbrmiR397a*, respectively. *NbActin* served as a housekeeping gene for lignin biosynthesis genes.

Reverse transcription was performed using one‐step gDNA removal and cDNA synthesis kit (Transgen, Beijing, China). Q‐RT‐PCR analysis was performed using a thermocycler. *NbActin* served as the housekeeping gene for *NbLAC*s.

### Computational prediction and experimental validation of *PbrmiR397a* targets

*PbrmiR397a* targets were predicted from the pear and tobacco genome sequences using the psRNATarget server (Dai and Zhao, 2011). A penalty score of 2.5 calculated as previously described (Jiu *et al*., 2015; Leng *et al*., 2016) was applied to potential target sites in the miRNA:mRNA duplexes (Figure S2b). Experimental validation of predicted targets was carried out using a modified RNA ligase‐mediated 5′‐RACE and a GeneRacer kit (Thermo Fisher Scientific, Massachusetts, USA). PCR was performed on cDNA from ‘Dangshansuli’ pear fruit at 21 DAF and stem tissue of 2‐month‐old tobacco plants. Primers used for amplifying are shown in Table S8. The PCR products were gel purified, subcloned into pEasy vectors (Transgen) and sequenced using the plasmid DNA of at least eight independent clones.

The pGreenII Dual‐Luciferase miRNA Target Expression Vector, derived from pGreenII 0800‐LUC (Hellens *et al*., 2005), was used to quantitatively evaluate miRNA activity. For this evaluation, an effector and three control vectors were constructed to contain CaMV35S::miR397a, CaMV35S::LUC, 35S::LUC::5× ts‐miR397 and 35S::LUC::5× its‐miR397, respectively (Figure 3b). In the positive control vector 35S::LUC::5× ts‐miR397, LUC was fused to five copies of target sequence of *PbrmiR397a*. In the negative control vector 35S::LUC::5× its‐miR397, LUC was fused to five copies of inverted target sequence of *PbrmiR397a*. The candidate miR397a target and its flanking sequence (200‐ to 300‐bp upstream and downstream of target sequence) were PCR amplified from cDNA of ‘Dangshansuli’ pear fruit at 21 DAF and stem tissue of 2‐month‐old tobacco plants using primers designed to bind to *LAC* genes of pear and tobacco (Table S8). Candidate target vectors were constructed by fusing these PCR fragments to the 3′ of the LUC gene in the basal plasmid (35S::LUC) using ClonExpress Entry One Step Cloning Kit (Vazyme, Nanjing, China). The mutant candidate target and its flanking sequence were amplified with alterations of two bases (GC to AA) at cleavage site of the target sequence using the method described previously (Nicolas, 2011; Yan and Cullen, 2003) and primers listed in Table S8. Renilla luciferase (REN) was in the same vector as LUC and was used as an internal control for normalization of LUC expression.

All the vectors were transferred into *A. tumefaciens* GV3101. *Agrobacterium* containing the effector vector or report vector were cultured separately and then mixed in a ratio of 10:1 in an infiltration buffer (10 mm MgCl_2_; 200 μm acetosyringone; 10 mm MES, pH 5.5) to a final concentration of OD_600_ between 0.7 and 1.0. Young *N. benthamiana* leaves were infiltrated with the mixture of *Agrobacterium* cultures using needleless syringes. The *N. benthamiana* plants were grown in a glasshouse with daylight extension to 16 h. For each effector–reporter combination, two independent experiments were performed using three infiltrated leaves from three different plants in each experiment. Three days after infiltration, LUC and REN were assayed using dual‐luciferase assay reagents (Promega, Madison, USA). The relative ratio of LUC/REN calculated and compared to the corresponding control value set at 100.

### Functional study of *PbrmiR397a* and *PbrLAC* using transiently transformed pear fruit flesh

We prepared a pCambia 1301 construct to overexpress *PbrmiR397a* (35S‐*PbrmiR397a*; Figure S8b). Three unique parts of *PbrLAC1*,* 2* and *18* were inserted into the vector in a reverse orientation to form the antisense constructs 35S‐anti*PbrLAC1*, 35S‐anti*PbrLAC2*, and 35S‐anti*PbrLAC18* (Figure S8b). These constructs and EVs were individually transformed into *A. tumefaciens* GV3101. For infiltration, GV3101 cells were inoculated in Luria‐Bertani (LB) medium and cultured at 28 °C while shaking at 200 r.p.m. for 1 day. Afterwards, these cells were centrifuged, resuspended in an infiltration buffer (10 mm MgCl_2_; 200 μm acetosyringone; 10 mm MES, pH 5.5) and then maintained at 21 °C for 6 h. The cells were infiltrated into the flesh of ‘Dangshansuli’ fruit at 35 DAF using needleless syringes. Six fruits were injected with each construct in an experiment that was repeated three times. The transformed fruit was placed in the dark at 21 °C overnight and then transferred to a growth chamber (21 °C, 16‐h light/8‐h dark photoperiod) under low light conditions for 10 days before being examined and imaged (Zhou *et al*., 2015).

### Tobacco plant transformation and lignin analysis

The overexpression vector *PbrmiR397a* used in fruit infiltration was also used for tobacco (*N. tabacum*) transformation via the *Agrobacterium*‐mediated leaf disc transformation method (Thomashow, 1999).

Nine randomly selected hygromycin‐resistant independent T0 transgenic tobacco plants were analysed by PCR using *PbrmiR397a*‐specific primers (Table S8) to confirm the presence of *PbrmiR397a* transgene. Seeds of a T0 plant were sown on hygromycin selection medium to generate T1 plants. Ten T1 plants from a T0 were planted in a greenhouse for seed collection from each plant separately. These seeds were sown on hygromycin containing medium to generate T2 plants. If 100% of T2 plants were hygromycin resistance, the T1 plant was considered as a homozygous transgenic plant and its seeds were resown directly into soil without hygromycin selection to generate T2 plants that were used for the subsequent experiments. Transcript levels of *PbrmiR397a* in SDX at the second internode from the shoot tips of 8‐week‐old T2‐generation plants were analysed by q‐PCR using primers described in Table S8. The experiment was repeated for three biological samples from three different plants. Four of these lines (397a‐3, 397a‐4, 397a‐8 and 397a‐11) that exhibited relatively high levels of *PbrmiR397a* expression were used for the subsequent experiments. Plants of each line were grown in a glasshouse with daylight extension to 16 h. Plant height, fresh weight of leaves per plant and whole plant, and leaf area were determined for eight plants of each transgenic line and WT control when the plants were at 16 weeks old.

Whole stems from WT and T2 transgenic plants were frozen in liquid nitrogen. After freezing, the xylem ring from five plants of each line and WT was manually collected by removing the pith and cortex. The xylem tissues were pooled from five plants, milled to a fine powder and sequentially extracted with acetone, a mix of toluene and ethanol (2/1, v/v), ethanol, and water. The resulting cell wall residue was dried for lignin analysis. Klason lignin content was estimated according to the standard procedures (Lin and Dence, 2012). The lignin composition was studied by thioacidolysis, as previously described (Lapierre *et al*., 1995). The lignin‐derived thioacidolysis monomers were identified by GC‐MS as their trimethylsilylated derivatives. All the results were the mean value obtained from four independent experiments performed on the same amount of starting materials derived from pooled xylem tissues of five plants.

### Microscopy

The second highest internode of 8‐week‐old tobacco stems from five plants of WT and T2‐generation *PbrmiR397a* transgenic lines were fixed in formalin‐acetic acid‐alcohol fixative at 4 °C for 1 week. Samples were dehydrated in an ethanol series and embedded in paraffin. Cross‐sections (5 μm) were cut with a Leica RM 2015 ultramicrotome (Leica Microsystems, Wetzlar, Germany). The sections were placed onto glass slides and stained with 1% (w/v) Toluidine Blue O [with 1% (w/v) sodium borate] for 5 min, after which the sections were observed using a Leica TCs SP2 spectral confocal microscope (Leica Microsystems). The width of xylem in WT and transgenic plants was measured with the images of Toluidine Blue O‐stained sections by Image‐Pro Plus software. The width of xylem from each line was the mean value of eight sections at the diagonal position in the image of the stem cross‐section from five plants of each lines.

For visualization of total lignin autofluorescence, 5‐μm sections were observed using a Leica TCS SP2 AOBS confocal laser scanning microscope (Leica Microsystems) illuminated with a 405‐nm blue diode laser, and emission was detected at 460 nm (Vanholme *et al*., 2013). To obtain comparable images, laser intensity, pinhole, and photomultiplier gain settings were kept constant between samples.

Regarding transmission electron microscopy (TEM), the same materials of paraffin section were fixed in fixative for 12 h. The method was used as described previously (Whitehill *et al*., 2016). The secondary wall thickness of vessel cells was measured with the images of TEM. Five TEM images were selected randomly from each line, and eight values of cell wall thickness at the diagonal position were obtained from each image, after which the final mean values of five images were calculated for each line.

### SNP calling, cis‐element predicting and phylogenetic analysis

Sixty varieties were collected from the Chinese National Pear Germplasm Repository in Wuhan. The sequences of 150 bp of paired‐end reads were then filtered with Trimmomatic software (Bolger *et al*., 2014). The total clean data for each sample were at least 5.3 Gb (10× genome coverage). Sequence read mapping (default parameters) was carried out using Burrows‐Wheeler alignment (Li and Durbin, 2009). The module ‘mpileup’ in SAMtools (Li *et al*., 2009) was used for SNP calling. An in‐house Perl script was then used to analyse the effectiveness of the SNPs. The lowest number of reads to support the SNPs was four, and the individual base percent was greater than 25%. Cis‐elements in promoter region were predicted with PlantCARE (Rombauts *et al*., 1999). Phylogenetic analysis was performed based on sequence data of the 3.0‐kb promoter region of the *PbrmiR397a* gene.

### Dual‐luciferase assays of transiently transformed leaf protoplasts

The 3.0‐kb promoter region of the *PbrmiR397a* gene was amplified from ‘Chenjiadamali’ (HG pear variety) and ‘Cuilv’ (LG pear variety) and inserted into a pGreenII 0800‐LUC vector (Hellens *et al*., 2005). Four partial promoter sequences of the *PbrmiR397a* gene from the LG (pmiR397a‐LG1, pmiR397a‐LG1m, pmiR397a‐LG‐2 and pmiR397a‐LGD) were amplified and inserted into the same vector (see Figure 5d). Primers used for amplifying are shown in Table S8. The transformed colonies were selected and cultured for plasmid DNA that was purified with a Plasmid Maxprep Kit (Vigorous, Beijing, China). The plasmid DNA of reporter constructs (promoter‐luciferase) was transferred into the protoplasts from leaves of 4‐week‐old short‐day *Arabidopsis* plants as previously described (Yoo *et al*., 2007). After transfection, protoplasts were cultured for 18 h. The method used for hormone treatment as previously described with modifications (Wang *et al*., 2016). A series of concentrations of auxin and SA were applied to each sample at 2 h after transfection. Luciferase activities were determined using a dual‐luciferase reporter assay system (Promega). Relative luciferase activities of treatment samples were calculated by normalizing against the activity of control samples.

### Statistical analysis

The data were statistically processed using the SAS software package (SAS Institute, North Carolina, USA); analysis of variance was used to compare the statistical difference based on *t*‐test at the significance levels of *P *< 0.05 (*), *P* < 0.01 (**).

## Conflict of interest

The authors declare no conflict of interests.

## Data accessibility

The genome sequences of the 60 pear varieties are available at our professional website (http://peargenome.njau.edu.cn).

## Accession numbers

Sequence data in this article can be found at NCBI with the following accession numbers. *PbrmiR397a* (KY438934); *PbrLAC1* (KY438931); *PbrLAC2* (KY438932); *PbrLAC18* (KY438933), pmiR397a (HG) (KY438935) and pmiR397a(LG) (KY438936).

## Online resources

Arabidopsis laccase protein sequences (TAIR, www.arabidopsis.org/).

Pear genome sequences (http://peargenome.njau.edu.cn/).

Apple, peach and strawberry genome sequences (https://www.rosaceae.org).

Tobacco genome sequences (www.solgenomics.net/).

tBLASTn and BLASTp algorithms (www.ncbi.nlm.nih.gov/BLAST).

National Center for Biotechnology Information conserved domain database (www.ncbi.nlm.nih.gov/Structure/cdd/wrpsb.cgi).

In‐house Perl script (https://github.com/Sunhh/NGS_data_processing).

## Supporting information


**Figure S1** Subcellular localization of PbrLACs in onion epidermal cells.

**Figure S2** *PbrmiR397a* and its target genes.

**Figure S3** Relative activity levels of luciferase. *Nicotiana benthamiana* leaves were transfected with reporter constructs, but without PbrmiR397a construct. Data are means ± SD (*n* = 6).

**Figure S4** Second biological repeats of transient assays using *PbrmiR397a* overexpression and *PbrLAC1*,* PbrLAC2* and *PbrLAC18* antisense constructs in ‘Dangshansuli’ fruit at 35 DAF. Black arrows show the infiltration sites.

**Figure S5** Multiple sequence alignment of the nucleotide sequence of the 3.0‐kb promoter of seven pear varieties. ‘Dangshansuli’ is the reference sequence. ‘Chenjiadamali’, ‘Liyuanchentuoli’ and ‘Xingshan No. 29’ are HG varieties, whereas ‘Biyun No. 15’, ‘Cuilv’ and ‘Taiwanchihua’ are LG varieties.

**Figure S6** Results of the protoplast transient assay.

**Figure S7** q‐RT‐PCR expression profiles of 20 *LAC* genes in the SDX of WT tobacco plants grown under long‐day conditions. M, DNA marker.

**Figure S8** Schematic diagram of gene constructs.

**Table S1** Identification of *LAC* genes in the *Pyrus bretschneideri*,* Malus domestica*,* Fragaria vesca*,* Prunus persica*,* Nicotiana tabacum,* and *Arabidopsis thaliana*.

**Table S2** RPKM (Reads Per Kilobases of transcript per Million mapped reads) values of *LAC* genes during the six stages of fruit development in six pear cultivars.

**Table S3** RPKM values of *LAC* genes during fruit development in two apple cultivars.

**Table S4** RPKM values of *LAC* genes during the four stages of fruit development in three peach cultivars.

**Table S5** RPKM values of *LAC* genes during the seven stages of strawberry fruit development.

**Table S6** Correlation analysis between SNPs of *PbrLAC*s and stone cell content in 60 pear varieties.

**Table S7** Relative expression levels of lignin biosynthetic genes between PbrmiR397a overexpressing and wild‐type tobacco plants as determined by q‐PCR.

**Table S8** Primers used in this study.
