# Supplementary material for: PbrmiR397a regulates lignification during stone cell development in pear fruit
Source: Plant Biotechnol J. 2018 Jun 21;17(1):103–17. doi: 10.1111/pbi.12950 (PMC6330545; doi:10.1111/pbi.12950)
Supplement: Supplementary file 1 — Figure S1 Subcellular localization of PbrLACs in onion epidermal cells. [file PBI-17-103-s001.pdf]

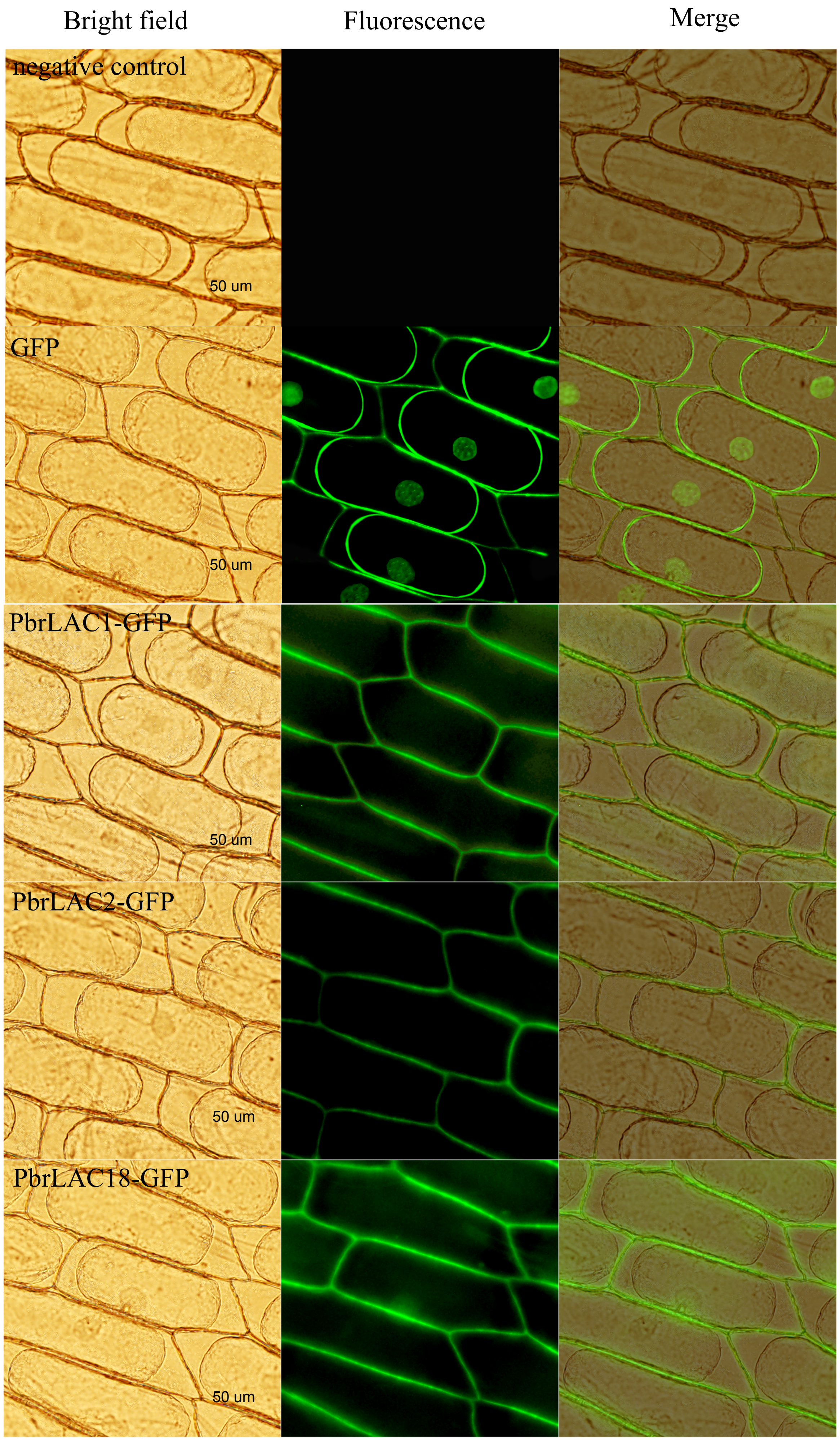

Figure S1 Subcellular localization of PbrLACs in onion epidermal cells.

All genes were under the control of the CaMV 35S promoter.
