# Supplementary material for: PbrmiR397a regulates lignification during stone cell development in pear fruit
Source: Plant Biotechnol J. 2018 Jun 21;17(1):103–17. doi: 10.1111/pbi.12950 (PMC6330545; doi:10.1111/pbi.12950)
Supplement: Supplementary file 2 — Figure S2 PbrmiR397a and its target genes. [file PBI-17-103-s014.pdf]

(a)

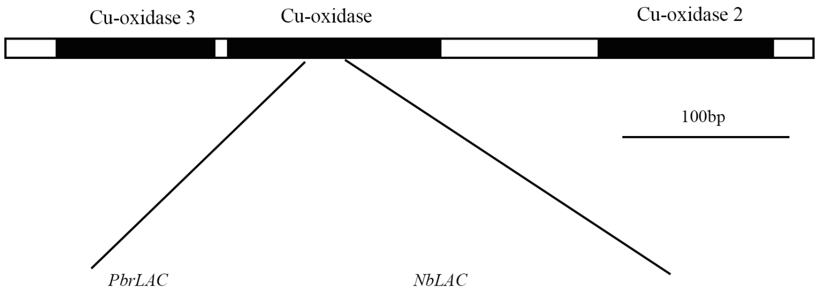

PbrLAC		*NbLAC*	
PbrLAC1	CATCAATGCTGCACTCAATGA	*NbLAC1*	CATCAATGCTGCACTCAATGA
PbrLAC2	CATCAATGCTGCGCTCAATGA	*NbLAC2*	GATCAATGCTGCGCTCAATGA
PbrLAC3	CATCAATGCTGCACTGAATGA	*NbLAC3*	CATCAACGCTGCACTCAATGA
PbrLAC4	CATCAATGCTGCACTAAACGA	*NbLAC5*	CGTCAATGCTGCTCTCAATGA
PbrLAC5	CATCAATGCTGCACTAAACGA	*NbLAC7*	TGTCAACGCTGCTCTCAATGA
PbrLAC6	CATCAATGCTGCACTCAATGA	*NbLAC8*	AGTCAATGCTGCACTGAATGA
PbrLAC7	CATCAATGCTGCACTCAATGA	*NbLAC9*	AGTCAATGCTGCACTGAATGA
PbrLAC8	CATTAATGCTGCACTCAATGA	*NbLAC10*	GATCAATGCTGCACTCAATGA
PbrLAC14	AATCAACGCAGCACTCAACGA	*NbLAC11*	GATCAATGCTGCACTCAATGA
PbrLAC15	AATCAACGCTGCGCTCAATGA	*NbLAC12*	GATCAATGCTGCACTCAATGA
PbrLAC16	GATCAACGCCGCTCTCAATGA	*NbLAC14*	AATCAATGCTGCACTCGATGA
PbrLAC17	GATCAACGCCGCACTCAATGA	*NbLAC15*	AATCAATGCTGCACTCAATGA
PbrLAC18	GATCAACGCTGCAGTCAATGA	*NbLAC16*	AATCAATGCTGCACTCAATGA
PbrLAC19	GATCAACGCCGCTCTCAATGA	*NbLAC17*	GGTCAATGCTGCACTTAATGA
PbrLAC20	GATCAACGCCGCTCTCAATGA	*NbLAC18*	TGTCAATGCTGCTCTCAATGA
PbrLAC21	CATCAACTCTGCACTCAATCA	*NbLAC19*	CATCAATGCTGCACTCAATAA
PbrLAC22	TATCAATGCAGCACTCAACGA	*NbLAC20*	CATCAATGCTGCACTCAATAA
PbrLAC23	AATCAACGCTGCACTCAATAC	*NbLAC24*	AATCAATGCTGCACTCAATCA
PbrLAC24	AATCAATGTTGCACTCAATAA	*NbLAC29*	AGTCAATGCTGCACTGAATGA
PbrLAC29	AATCAACGCTGCACTCAATAA	*NbLAC32*	TATCAATGCTGCACTCAATCA
PbrLAC30	AATCAACGCTGCACTCAATAA		
PbrLAC33	CATCAATGCCGCCCTCAACCA		
PbrLAC36	CGTCAACTCTGCACTCAATCA		
PbrLAC37	TATCAACGCAGCACTCAACGA		

(b)

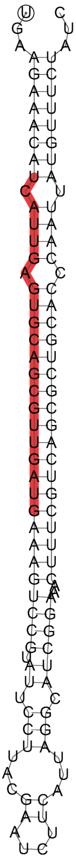

Figure S2 *PbrmiR397a* and its target genes.
(a), Twenty-four *PbrLAC*s and twenty *NbLAC*s are targets of *PbrmiR397a*.
(b), Secondary structure of the *PbrmiR397a* precursor. The sequence of *PbrmiR397a* is highlighted in red.
