# Supplementary material for: PbrmiR397a regulates lignification during stone cell development in pear fruit
Source: Plant Biotechnol J. 2018 Jun 21;17(1):103–17. doi: 10.1111/pbi.12950 (PMC6330545; doi:10.1111/pbi.12950)
Supplement: Supplementary file 3 — Figure S3 Relative activity levels of luciferase. Nicotiana benthamiana leaves were transfected with reporter constructs, but without PbrmiR397a construct. Data are means ± SD (n = 6). [file PBI-17-103-s013.pdf]

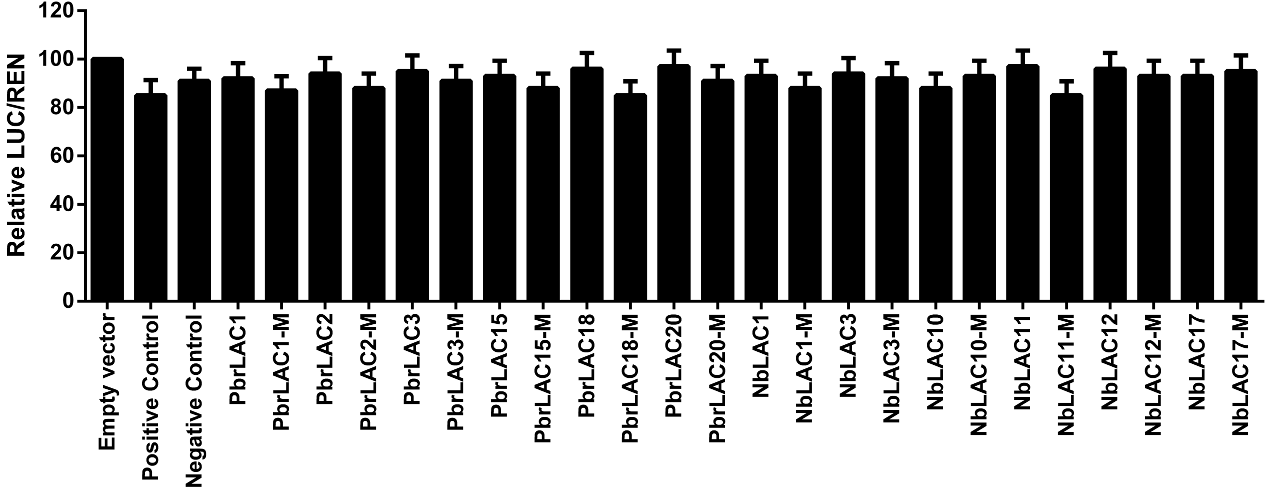

Figure S3 Relative activity levels of luciferase. *N. benthamiana* leaves were transfected with reporter constructs, but without *PbrmiR397a* construct. Data are means ± SD (n = 6).
