# Supplementary material for: PbrmiR397a regulates lignification during stone cell development in pear fruit
Source: Plant Biotechnol J. 2018 Jun 21;17(1):103–17. doi: 10.1111/pbi.12950 (PMC6330545; doi:10.1111/pbi.12950)
Supplement: Supplementary file 4 — Figure S4 Second biological repeats of transient assays using PbrmiR397a overexpression and PbrLAC1, PbrLAC2 and PbrLAC18 antisense constructs in ‘Dangshansuli’ fruit at 35 DAF. Black arrows show the infiltration sites. [file PBI-17-103-s015.pdf]

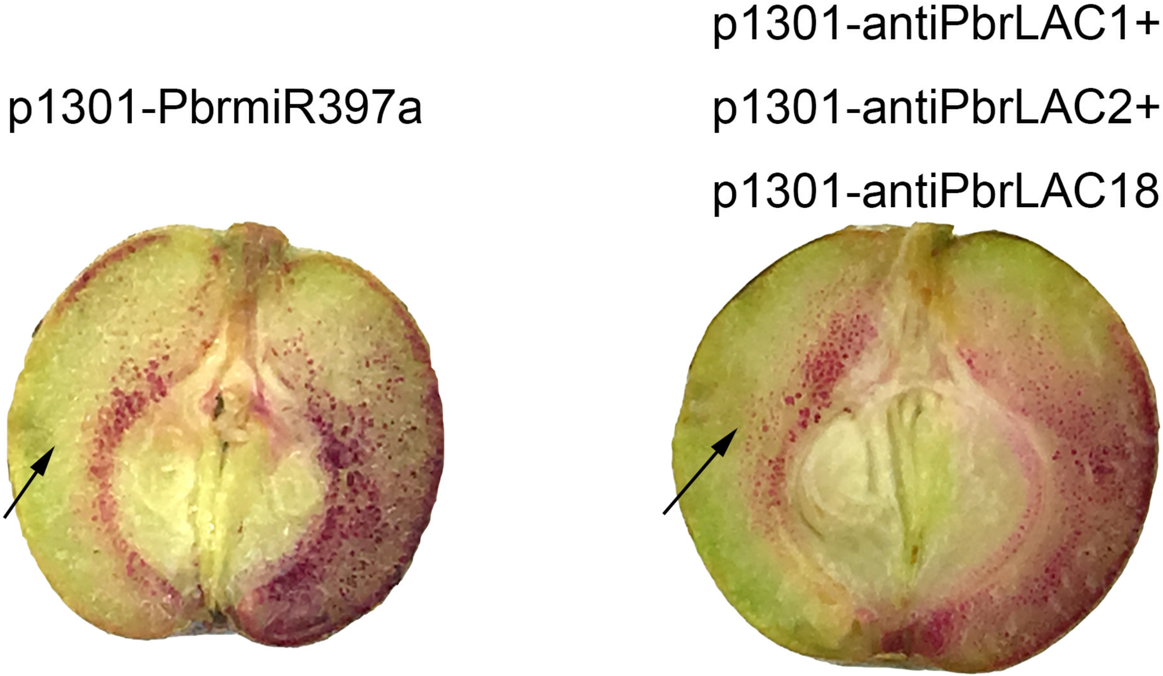

Figure S4 Second biological repeats of transient assays using *PbrmiR397a* overexpression and *PbrLAC1*, *PbrLAC2* and *PbrLAC18* antisense constructs in ‘Dangshansuli’ fruit at 35 DAF. Black arrows show the infiltration sites.
