# Supplementary material for: PbrmiR397a regulates lignification during stone cell development in pear fruit
Source: Plant Biotechnol J. 2018 Jun 21;17(1):103–17. doi: 10.1111/pbi.12950 (PMC6330545; doi:10.1111/pbi.12950)
Supplement: Supplementary file 6 — Figure S6 Results of the protoplast transient assay. [file PBI-17-103-s002.pdf]

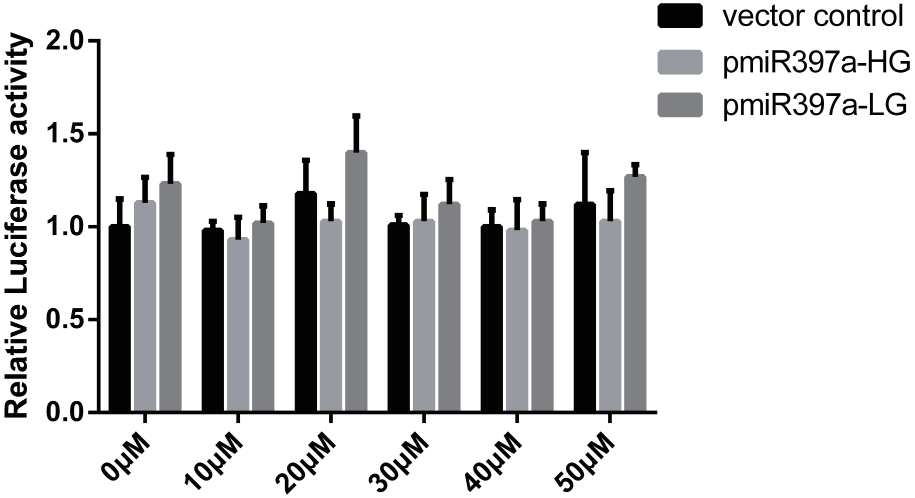

Figure S6 Results of the protoplast transient assay.
Relative luciferase activity was driven by 3.0-kb *PbrmiR397a* promoter fragments from the HG and LG. Each sample was treated with a series of concentrations of auxin. The values are presented as the means±SEs. Three biological repeats showed the same trend.
