# Supplementary material for: PbrmiR397a regulates lignification during stone cell development in pear fruit
Source: Plant Biotechnol J. 2018 Jun 21;17(1):103–17. doi: 10.1111/pbi.12950 (PMC6330545; doi:10.1111/pbi.12950)
Supplement: Supplementary file 7 — Figure S7 q‐RT‐PCR expression profiles of 20 LAC genes in the SDX of WT tobacco plants grown under long‐day conditions. M, DNA marker. [file PBI-17-103-s003.pdf]

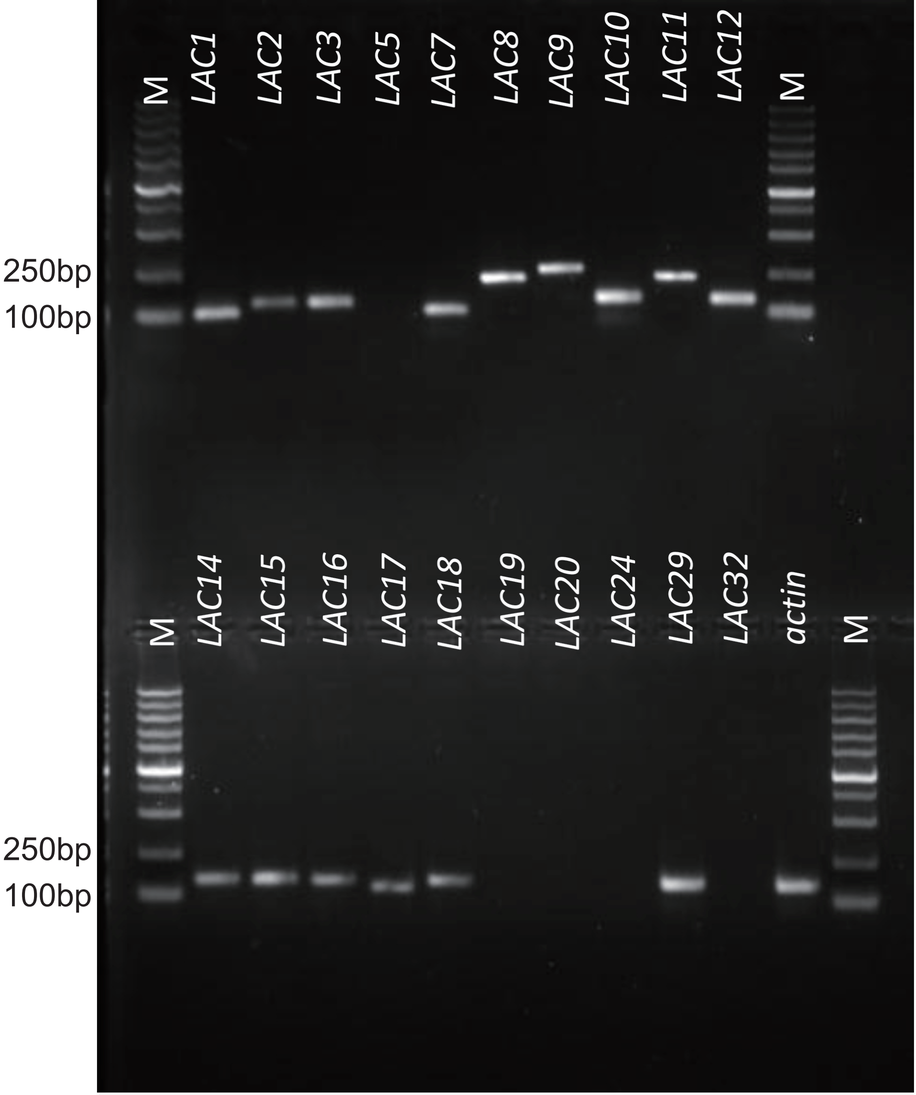

Figure S7 q-RT-PCR expression profiles of 20 *LAC* genes in the SDX of WT tobacco plants grown under long-day conditions. M, DNA marker.
