# Supplementary material for: PbrmiR397a regulates lignification during stone cell development in pear fruit
Source: Plant Biotechnol J. 2018 Jun 21;17(1):103–17. doi: 10.1111/pbi.12950 (PMC6330545; doi:10.1111/pbi.12950)
Supplement: Supplementary file 8 — Figure S8 Schematic diagram of gene constructs. [file PBI-17-103-s004.pdf]

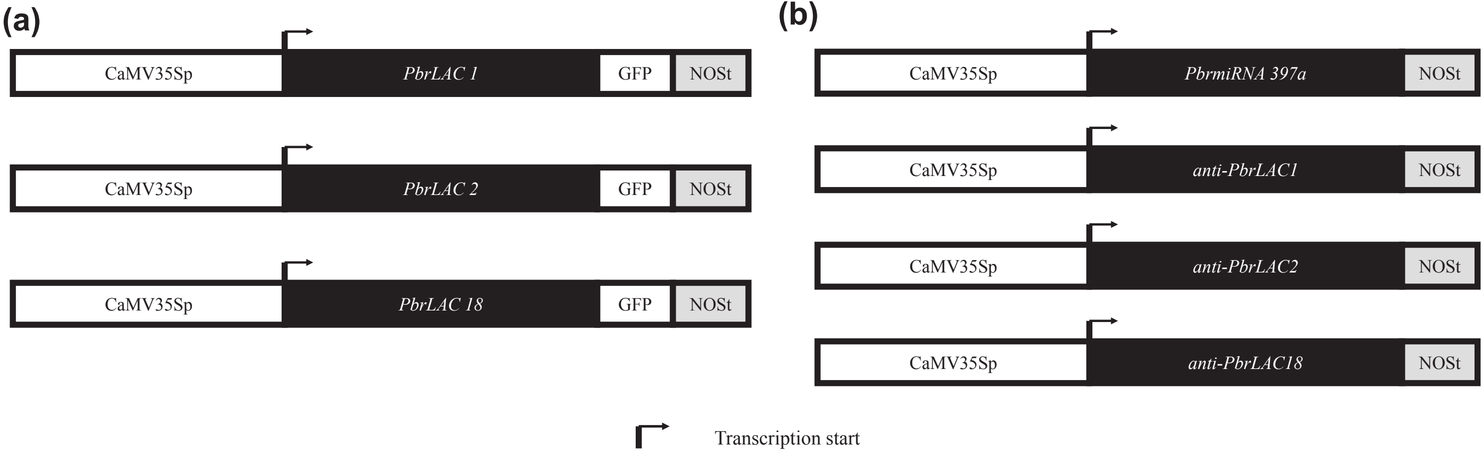

Figure S8 Schematic diagram of gene constructs.
(a), Constructs used for the subcellular localization of genes.
(b), Constructs used for the agro-infiltration of pear fruit.
